# The Design and Investigation of Nanocomposites Containing Dimeric Nematogens and Liquid Crystal Gold Nanoparticles with Plasmonic Properties Showing a Nematic-Nematic Phase Transition (N_u_-N_x_/N_tb_)

**DOI:** 10.3390/ma7053494

**Published:** 2014-04-30

**Authors:** Maria-Gabriela Tamba, Chih Hao Yu, Bai Jia Tang, Christopher Welch, Alexandra Kohlmeier, Christopher P. Schubert, Georg H. Mehl

**Affiliations:** Department of Chemistry, University of Hull, Hull, HU6 7RX, UK; E-Mails: gabriela_tamba@yahoo.com (M.-G.T.); dearchyu@hotmail.com (C.H.Y.); b.tang@2005.hull.ac.uk (B.J.T.); c.welch@hull.ac.uk (C.W.); alex_km@yahoo.de (A.K.); chris.schubert@chem.queensu.ca (C.P.S.)

**Keywords:** nanocomposite, liquid crystal, nematic-nematic, phase diagram, dimer, siloxane, mixtures, nanoparticles, plasmonic, gold

## Abstract

The construction of liquid crystal compositions consisting of the dimeric liquid crystal, **CB_C9_CB** (cyanobiphenyl dimer = 1″,9″-bis(4-cyanobiphenyl-4′-yl)nonane), and the range of nematic systems is explored. The materials include a laterally functionalized monomer, which was used to construct a phase diagram with **CB_C9_CB**, as well as one laterally linked dimer liquid crystal material and two liquid crystal gold nanoparticle (LC-Au-NPs) systems. For the Au-NP-LCs, the NP diameters were varied between ~3.3 nm and 10 nm. Stable mixtures that exhibit a nematic-nematic phase transition are reported and were investigated by POM (polarizing optical microscopy), DSC (differential scanning calorimetry) and X-ray diffraction studies.

## 1. Introduction

The investigation of materials that show nematic-nematic transitions has increased considerably over the last few years since the first report on such a phase behaviour in dimeric liquid crystals [1]. This is due to the scientific novelty of a new nematic phase, different in organisation to that of the classical uniaxial nematic (Nu) phase. Additionally, there is considerable interest in the structure of this low temperature nematic phase, as its mode of assembly is not yet fully understood. Hence, it has been identified, rather cautiously, as N_x_, as a twist-bent nematic phase, N_tb_, or as a heliconical nematic phase [2–10]. These issues of phase assignment still need to be clarified, but they raise the more general question of whether a number of different thermotropic nematic phases, positioned below the classical N phase, do exist in organic or inorganic materials [11–13]. Beyond the interest in the fundamental issues of phase structure, the observation that the low temperature nematic phase in dimers, which in this contribution is termed N_x_, simply because its structure has not yet been fully clarified, is characterized by the spontaneous formation of chiral domains. This is of technological relevance, as this property can, in principle, be used to either stabilize or to enhance chirality in already chiral systems, such as TGB (twist grain boundary) arrays, blue phases or to induce chirality in plasmonic metal nanoparticle systems [14]. Here, chirality promises a wide range of novel optical effects [15,16]. Moreover, these LC nanoparticle (NPs) systems are potentially extremely interesting for LC applications, as it has been shown that nanoparticles dispersed in liquid crystals can result in enhanced switching behaviour. It has been reported that electro-optical switching in the microsecond regime is possible in the N_x_ phase [9]. The first step to realize such advantageous properties is to explore the miscibility of metal nanoparticles in dimeric systems forming the N_x_ phase. This is the subject matter of this report. Here, we report the first miscibility study of gold nanoparticles (Au-NPs) in dimers with a nematic-nematic phase transition.

Though a number of NP systems have been functionalized with LC groups in order to enhance their solubility in liquid crystals, the number of calamitic nematogenic systems is very low, and the number of those that exhibit LC behaviour is very small indeed, as discussed in recent reviews [16–19].

## 2. Results and Discussion

**Figure 1.**
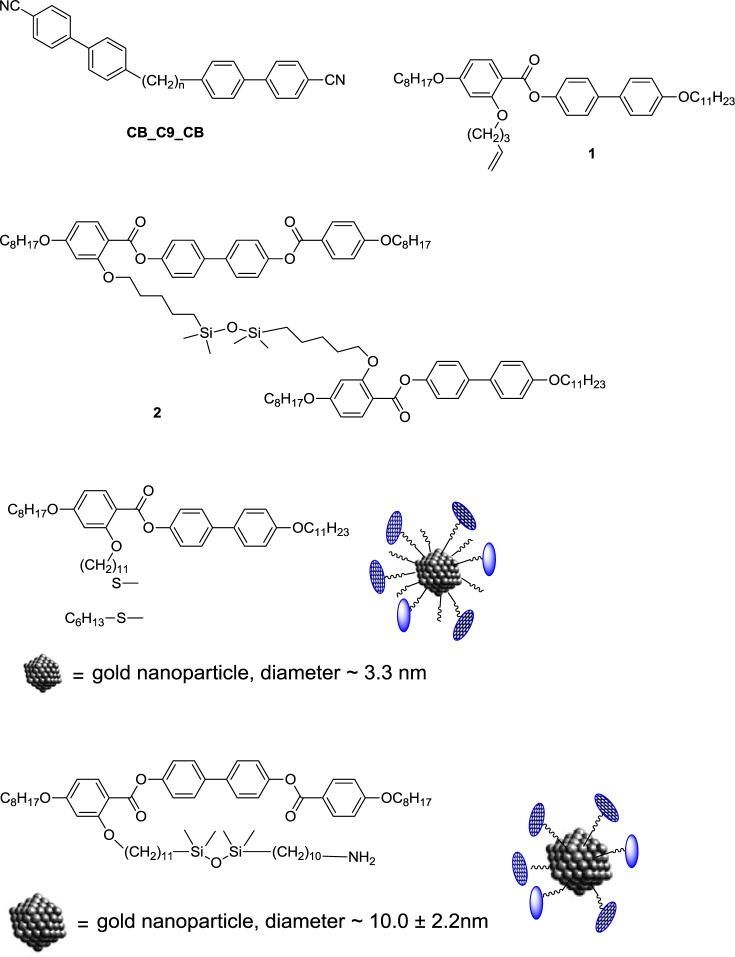
Chemical structures of the materials investigated. **CB_C9_CB**, cyanobiphenyl dimer.

As a starting point for the miscibility studies, the dimer, **CB_C9_CB** (cyanobiphenyl dimer), shown in Figure 1, was used, as the structure of the N_x_ phase in this material has been investigated intensively [1,7–10,20]. As LC-Au-NP systems, LC nanoparticle materials reported earlier were used [21–24]. As the LC-Au-NP materials to be investigated employ laterally connected mesogenic groups, different from those that have been shown to exhibit N_x_ phase behaviour, the design of the N_x_ phase forming composites was approached in a systematic study. In the first step, mixtures with a laterally functionalized nematogen, the liquid crystal, 4′-undecyloxybiphenyl-4-yl-4-octyloxy-2-(pent-4-en-1-yloxy) benzoate (**1**), shown in Figure 1, were performed in order to evaluate the limit of the phase stability in the N_x_ phase in such a mesogenic system. For that, a full phase diagram was constructed. A laterally functionalized mesogen was selected, due to the tendency of these systems to show nematic phase behaviour. In the second step, specific compositions of **CB_C9_CB** with a dimeric system containing siloxane groups (**2**) were prepared to gain additional knowledge in order to optimize the composition range for the mixtures with the LC-NPs. In the final step, compositions were prepared with LC gold particles. Here, liquid crystal Au-NPs were used, which have been reported earlier, using structures, very close to those of the laterally connected mesogen investigated initially. Two systems were explored.

In the first system, the particle size is as about 3.3 nm (Material **3**), and in the second material, the particle diameter is about 10 nm (Material **4**). The chemical structures of the investigated systems are shown in Figure 1.

**Figure 2.**
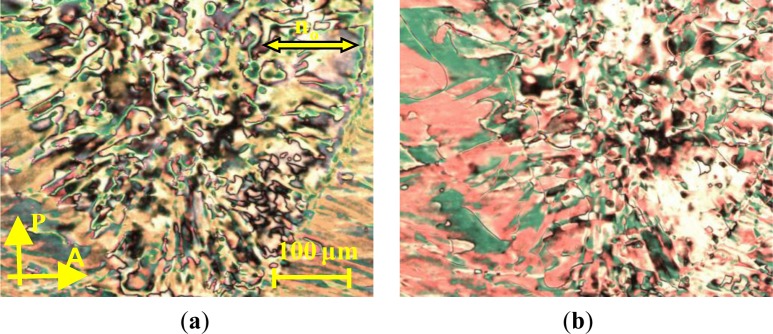
Textures of the nematic phase of **1** between untreated glasses observed between crossed polarizers. (**a**) N phase at 25 °C; (b) N phase at 50 °C.

The addition of the corresponding liquid crystal, **1** (4′-undecyl oxybiphenyl-4-yl 4-octyloxy-2- (pent-4-en-1-yloxy) benzoate), which has a positive dielectric anisotropy, Δε, to the dimer, **CB_C9_CB**, allows the construction of phase diagrams. Though the structures of **1** have been discussed earlier as a precursor for mesomorphic silsesquioxanes and carbosilazane dendrimers, detailed LC properties have not yet been discussed [25,26]. Compound **1** exhibits an enantiotropic nematic phase; typical defect textures are shown in Figure 2.

**Figure 3.**
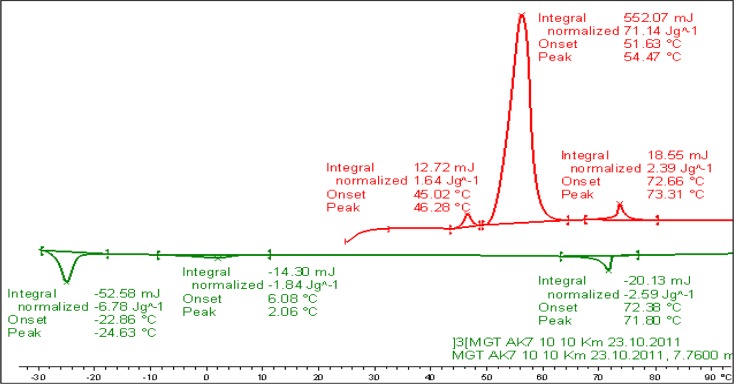
DSC studies (10 K/min) of Compound **1**; values are taken from the first heating and cooling scans. Cr 54.5 (71.14) N 73.3 (2.39) Iso (°C); Iso 71.8 (−2.59) N 2.06 (−1.84) X −24.6 (−6.78) Cr (°C); the numbers in brackets are the transition enthalpies in J·g^−1^.

DSC measurements were conducted at varying heating and cooling rates on samples of Compound **1**. The transition temperatures and the corresponding enthalpy values of Compound **1** taken from the heating and cooling DSC scan (10 K/min, see Figure 3) are given below.

**Figure 4.**
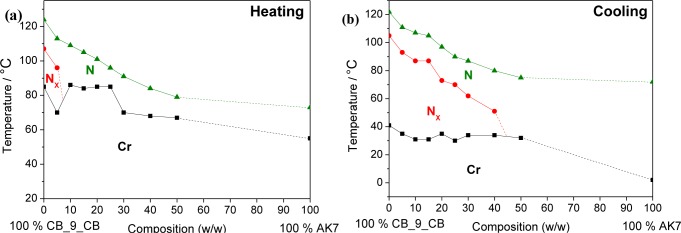
Transition temperatures of the binary mixtures of dimer **CB_C9_CB** in **1**, taken from the first DSC heating and cooling scans (10 K·min^−1^); the phase diagram of the transition temperatures of these mixtures, taken from the first DSC (**a**) heating scans (10 K·min^−1^) and (**b**) cooling scans (10 K·min^−1^).

A systematic investigation of the binary mixtures (w/w) of Material **1** with positive dielectric anisotropy with the cyanobiphenyl dimer, **CB_C9_CB** (5%–50% (w/w)), was carried out. All binary mixtures exhibit liquid crystalline behaviour. The mesophase types and the transition temperatures for these mixtures are given in Table 1. All compounds exhibit nematic phases. The mesophases of the mixtures were characterized by their optical textures, calorimetric studies and by X-ray investigations. No additional layer-structured mesophases are induced in the mixed phase region of this binary systems, *i.e.*, only nematic phases could be found. Remarkably, for the system, **CB_C9_CB**, more than 40% (w/w) of **1** can be added before the N_X_ phase is lost (see Figure 4). However, it is noted that the N_x_ phase stability is strongly reduced upon adding **1**, *i.e.*, for the system with 30% (w/w) of **1** in **CB_C9_CB** (**MGTP201**), a reduction of the transition temperature of the high-temperature N phase to the low-temperature N_x_ phase of about 54 °C could be found. In other words, the nematic N_X_ phase transition occurs on cooling at 62.1 °C. Moreover, the stability of the enantiotropic (that is thermodynamically stable) N_x_ phase is lost upon adding between 5% (w/w) to 10% (w/w) of the monomeric Compound **1**.

As a representative example the mesophase behaviour of the binary mixture, **MGTP201**, with 30% (w/w) **1** in **CB_C9_CB** will be described in more detail. The POM studies and the calorimetric investigations evidenced the existence of an N–N_x_ phase sequence with textural and structural features similar to the pure cyanobiphenyl dimer, **CB_C9_CB**.

**Figure 5.**
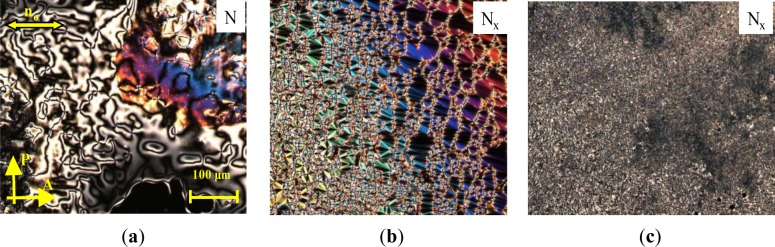
Textures of the **MGTP201** (30% (w/w) **1** in 70% **CB_C9_CB**) between untreated glasses observed in the same region on cooling between crossed polarizers: (**a**) schlieren texture in the high-temperature nematic N phase at 85 °C; (**b**) fan-like texture accompanied by rope texture developing in the low-temperature N_x_ phase at 48 °C and (**c**) sheared texture of the low-temperature N_x_ phase at 48 °C.

The high-temperature phase could be easily identified as a nematic phase by its characteristic texture. At the phase transition to the N_X_ phase, a fine structured rope texture or fan-like texture develops, as shown in Figure 5a,b. The sheared texture contains non-specific features with homeotropically oriented regions and some oily streaks, as shown in Figure 5c.

**Figure 6.**
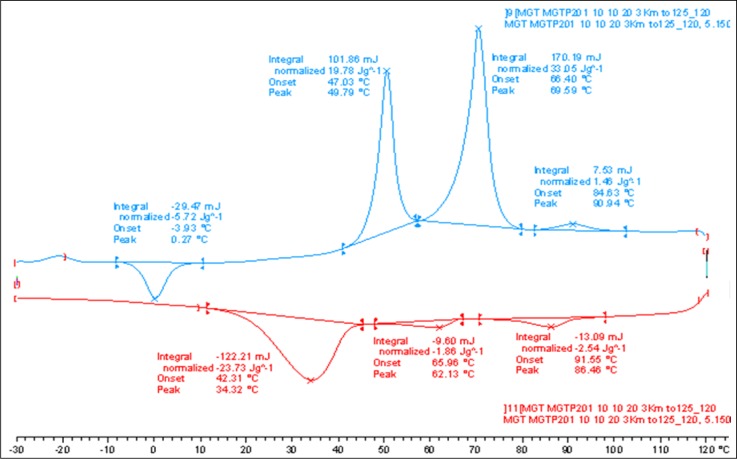
DSC studies (10 K/min) of the binary mixture, **MGTP201** (30% (w/w) **1** in 70% (w/w) **CB_C9_CB**); the values are taken from the second heating and cooling scans. Cr 69.6 (33.05) N 90.9 (1.46) Iso; Iso 86.5 (−2.54) N 62.1 N_x_ (−1.86) 34.2 (−23.73) Cr (°C); the numbers in brackets are the transition enthalpies in J·g^−1^.

DSC measurements were conducted at different heating and cooling rates on samples of the binary mixture, **MGTP201**, consisting of a mixture of 30% (w/w) **1** in **CB_C9_CB**. The transition temperatures and the corresponding enthalpy values of the compound **MGTP201** taken from the heating and cooling DSC scans recorded at 10 K/min (see Figure 6) are given below.

**Figure 7.**
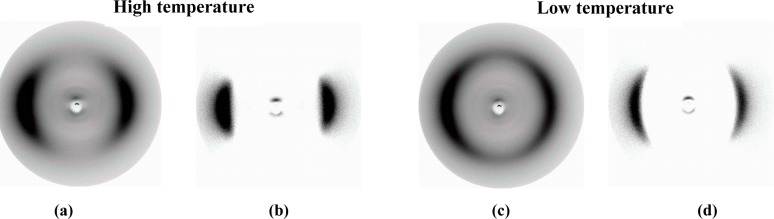
XRD patterns of an aligned sample of the mixture, **MGTP201**, in the magnetic field on cooling. (**a**,**b**) XRD patterns of the nematic phase at 77 °C: (**a**) original pattern; (**b**) the same XRD pattern, but the intensity of the isotropic liquid is subtracted; (**c**,**d**) XRD patterns of the N_x_ at 50 °C: (**c**) original pattern; (**d**) the same XRD pattern, but the intensity of the isotropic liquid is subtracted.

X-ray measurements were performed whilst applying a magnetic field of about 0.5 T to orient samples of the composition, **MGTP201**. The results confirm the presence of two nematic phases. The X-ray patterns in the high-temperature nematic state show the known features of the XRD patterns of the nematic phases of the pure cyanobiphenyl dimer, *i.e.*, diffuse crescent-like scattering on the meridian in the small angle region, parallel to the orienting magnetic field, and diffuse crescent-like scattering on the equator in the wide angle region. The diffractograms collected at 77 °C are shown in Figure 7a,b. Similar to the pure cyanobiphenyl dimer, this pattern does not change significantly at the phase transition to the low-temperature N_x_ phase recorded at 50 °C and shown in Figure 7c,d. There is no indication of a transition to a smectic phase, even though the textural features, such as the fan-shaped texture and or polygonal textures, would suggest, initially, the formation of a smectic-like structure.

It is noted that an addition of 15%–20% (w/w) of **1** to **CB_C9_CB** results in a decrease of the isotropisation temperature by ~20–24 °C, when compared to pure **CB_C9_CB**.

**Figure 8.**
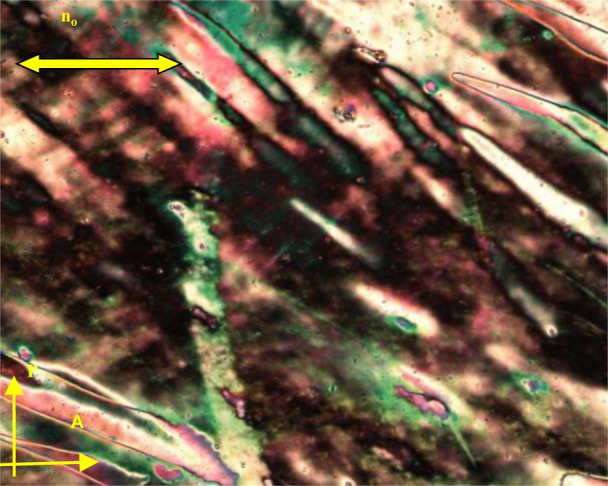
Textures of the nematic phase of **2** between untreated glasses observed between crossed polarizers.

Based on these successful miscibility studies with a monomer, the work was extended to the dimeric system, **2**, which is characterized by a very wide nematic range and by having features, such as the central siloxane group, that are present in one of the to be investigated LC gold nanoparticle systems. Compound **2** is a dimeric non-symmetric system, whose synthesis has been reported earlier [27]. Material **2** exhibits a wide enantiotropic nematic phase; a typical POM texture is shown in Figure 8. The nematic phase stability of **2** ranges on heating form −8.8 to 107.6 °C. The material was synthesized according to a reported method, and the results of the investigations of the LC phase behaviour are shown below [27].

**Figure 9.**
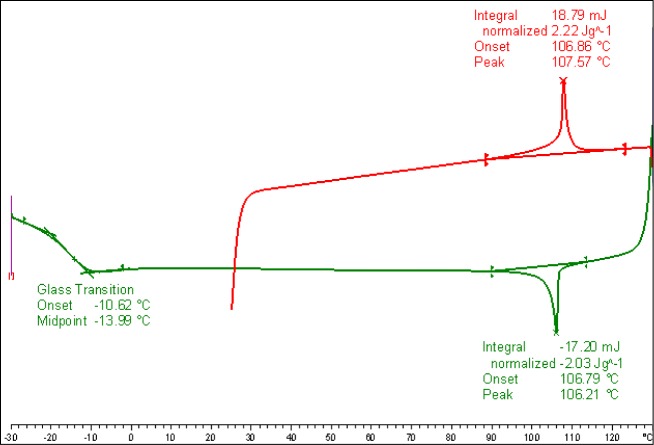
DSC studies (10 K/min) of Compound **2**, the first heating and cooling scan. The values are taken from the second heating and cooling scans. The transitions are: N 107.6 (2.22) Iso (°C); Iso 106.2 (−2.03) N −14.0 Tg (°C); the numbers in brackets are the transition enthalpies in J·g^−1^.

DSC measurements were conducted with different heating and cooling rates on samples of Compound **2**. The transition temperatures and the corresponding enthalpy values of Compound **2** taken from the heating and cooling DSC scans recorded at 10 K/min, shown in Figure 9, are given below.

An investigation of the selected composition of the binary mixtures (w/w) of Material **2** with a positive dielectric anisotropy with the cyanobiphenyl dimer, **CB_C9_CB**, at concentrations of 15% and 25% (w/w) evidenced that all of the compositions exhibit a nematic phase behaviour. The mesophase types and transition temperatures for these mixtures are given in Table 2. The mesophases of the mixtures were characterized by their optical defect textures, calorimetric studies and by X-ray investigations. No additional layer- structured mesophases are induced in the mixed phase region of this binary systems, *i.e.*, only nematic phases could be found.

**Figure 10.**
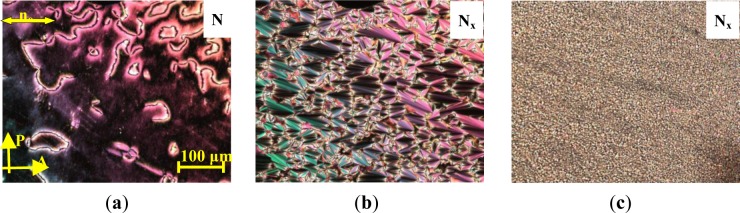
Textures of the **MGTP208** (15% (w/w) **2** in 85% (w/w) **CB_C9_CB**) between untreated glass slides, observed in the same region upon cooling between crossed polarizers: (**a**) schlieren texture in the high-temperature nematic N phase at 100 °C; (**b**) fan-like texture of the low-temperature N_x_ phase at 70 °C and (**c**) sheared texture of the low-temperature N_x_ phase at 70 °C.

It is noted that the recorded systems show monotropic N_x_ phase behaviour; or in other words, the N_x_ phase is a meta-stable phase, which can be obtained upon cooling from the nematic (N) phase. As a representative example of the mesophase behaviour, the results for the binary mixture, **MGTP208**, with 15% (w/w) **2** in **CB_C9_CB** will be described in more detail. The POM studies and the calorimetric investigations evidenced the existence of an N–N_x_ phase sequence with textural and structural features similar to the pure cyanobiphenyl dimer, **CB_C9_CB**. The high-temperature phase could be easily identified as a nematic phase by its characteristic texture; a typical schlieren texture is shown in Figure 10a. At the phase transition to the N_X_ phase, a fan-shaped texture develops. This is shown in Figure 10b. The sheared texture, shown in Figure 10c, contains non-specific features and some oily streaks.

**Figure 11.**
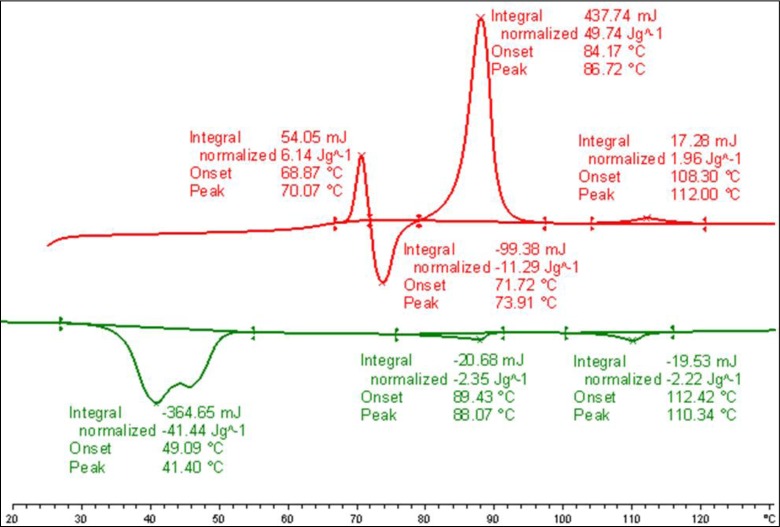
DSC studies (10 K/min) of the binary mixture, **MGTP208** (15% (w/w) **2** in 85% **CB_C9_CB**); the values are taken from the first heating and cooling scans. Cr 86.7 (49.74) N 112.0 (1.96) Iso (°C); Iso 110.3 (−2.22) N 88.1(−2.35) N_x_ 41.4 (−41.44) Cr (°C); the numbers in brackets are the transition enthalpies in J·g^−1^.

DSC measurements were conducted at different heating and cooling rates of samples of the binary mixture, **MGTP208**. The transition temperatures and the corresponding enthalpy values of the binary mixture, **MGTP208**, taken from the heating and cooling DSC scan collected at 10 K/min, are shown in Figure 11 below. The lowering of the isotropisation temperature of the composition of ~12 °C, when compared to pure **CB_C9_CB** is lower than found for the comparable mixture with the monomeric system, **1**.

**Figure 12.**
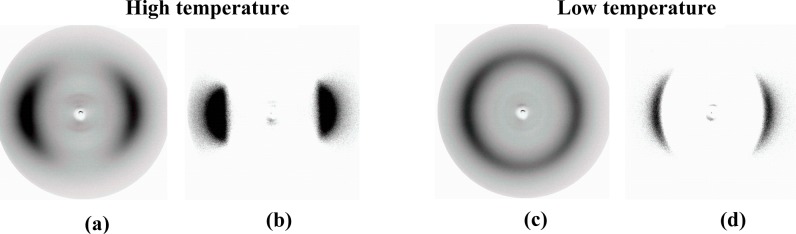
XRD patterns of an aligned sample of mixture **MGTP208** in the magnetic field upon cooling. (**a**,**b**) XRD patterns of the nematic phase at 97 °C: (**a**) original pattern; (**b**) the same XRD pattern, but the intensity of the isotropic liquid is subtracted. (**c**,**d**) XRD patterns of the N_x_ at 70 °C: (**c**) original pattern; (**d**) the same XRD pattern, but the intensity of the isotropic liquid is subtracted.

X-ray measurements were performed whilst applying a magnetic field of about 0.5 T applied to orient the samples of the composition, **MGTP208.** The results confirm the presence of two nematic phases. The X-ray patterns in the high-temperature nematic state show the known features of the XRD patterns of the nematic phase of the pure cyanobiphenyl dimer, *i.e.*, diffuse crescent-like scattering on the meridian in the small angle region (parallel to the orienting magnetic field) and diffuse crescent-like scattering on the equator in the wide-angle region. A diffractogram of the composition, **MGTP208**, recorded at 97 °C is shown in Figure 12a,b. Similar to the pure cyanobiphenyl dimer, this pattern does not change significantly at the phase transition to the low-temperature N_X_ phase. The diffractogram recorded at 70 °C is shown in Figure 12c,d. There is no indication of a transition to a smectic phase, even though the textural features, such as a fan-shaped texture and/or polygonal textures, occur.

**Figure 13.**
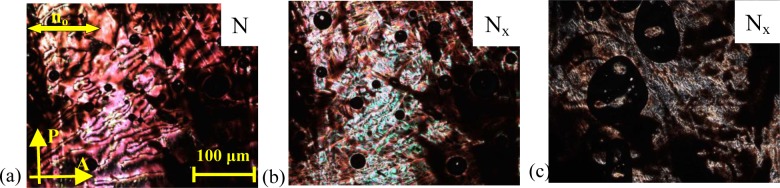
Textures of the binary mixture, **MGTP212** (15% (w/w) **3** in 85% **CB_C9_CB**), at *U* = 0 V between untreated glasses observed in the same region upon cooling between crossed polarizers: (**a**) schlieren texture in the high-temperature nematic N phase at 113 °C; (**b**) fan-like texture of the low-temperature N_x_ phase at 100 °C and (**c**) sheared texture of the low-temperature N_x_ phase at 84 °C.

Based on the results on the monomeric and dimeric systems, the miscibility of **CB_C9_CB** with liquid crystal gold nanoparticles was explored. These initial experiments suggested that a concentration of ~15% of the gold nanoparticle system in **CB_C9_CB** might still allow for N_x_ phase formation. As the initial LC gold nanoparticle system, Material **3** was selected. For **3**, the gold particles have a diameter of ~3.3 nm; the chemical synthesis, liquid crystal phase behaviour and plasmonic properties have been reported elsewhere [22]. The results for the mixture, **MGTP212** (15% **3** in 85% **CB_C9_CB** w/w) will be described. The POM studies and the calorimetric investigations evidenced the existence of the phase sequence, N–N_x_, with textural and structural features similar to the pure cyanobiphenyl dimer, **CB_C9_CB**. The high-temperature phase could be easily identified as a nematic phase by its characteristic texture. At the phase transition to the N_x_ phase, a fine structured fan-shaped texture develops, as shown in Figure 13a,b. The sheared texture contains non-specific features, a small-scale fan-shaped texture and homeotropically oriented regions and some oily streaks, shown in Figure 13c.

**Figure 14.**
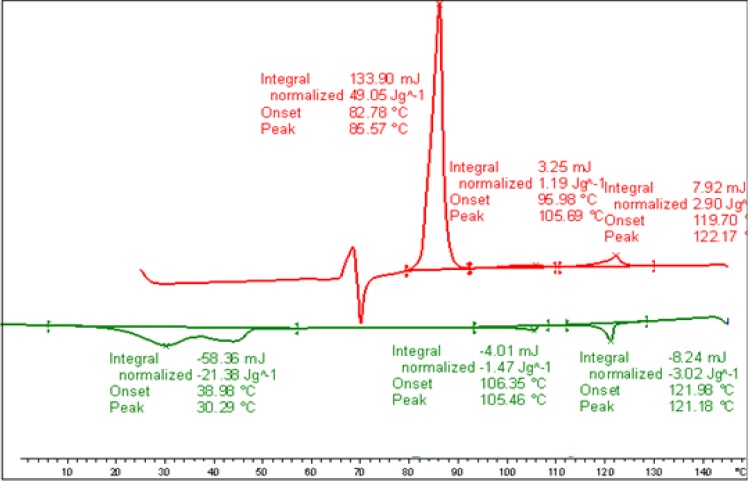
DSC studies (10 K/min) of the **MGTP212**; the values are taken from the first heating and cooling scans. Cr 85.6 (49.05) Nx 105.7 (1.19) N 122.2 (2.90) Iso (°C); Iso 121.2 (−3.02) N 105.5 (−1.47) Nx 30.3 (−21.38) Cr; the numbers in brackets are the transition enthalpies in J·g^−1^.

DSC measurements were conducted at different heating and cooling rates of samples of the binary mixture, **MGTP212** (15% **(**w/w) **3** in 85% **CB_C9_CB**). The transition temperatures and the corresponding enthalpy values of the binary mixture, **MGTP212**, taken from heating and cooling DSC scans (10 K/min) are shown in Figure 14. A crucial feature is that only a very small reduction of the isotropisation temperature occurs, when compared to pure **CB_C9_CB** and the stabilisation of the N_x_ phase formation, which is enantiotropic in this system can be observed.

**Figure 15.**
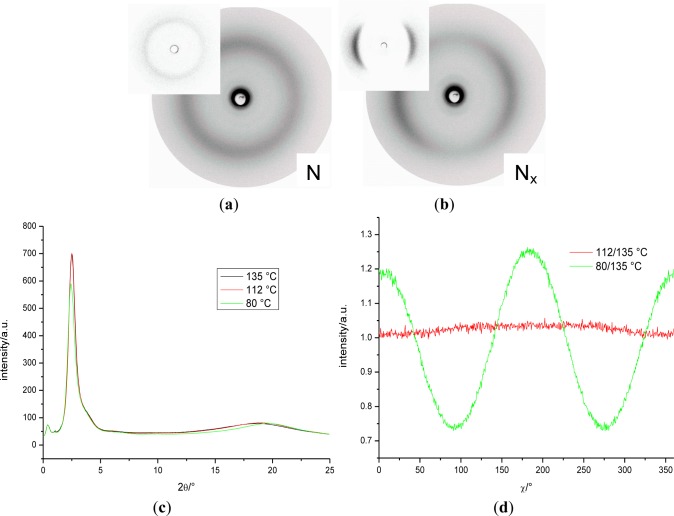
XRD patterns of an aligned sample of the mixture **MGTP212** in a magnetic field upon cooling: (**a**) N phase at 112 °C (the insert subtracted intensities were recorded in the isotropic phase); (**b**) N_x_ at 80 °C (the insert subtracted intensities of the isotropic phase); (**c**) θ-scan of the diffraction pattern in the N and N_x_ phases; SAXS: *d* = 0.470 nm (112 °C), *d* = 0.453 nm (80 °C); (**d**) distribution of the wide-angle scattering along χ in the N and N_x_ phases; intensity *vs*. χ (15°–25° 2θ).

X-ray measurements were performed whilst applying a magnetic field of about 0.5 T to orient the samples of the binary mixture, **MGTP212** (15% (w/w) **3** in 85% **CB_C9_CB**). The results confirm the presence of two nematic phases. The X-ray patterns in the high-temperature nematic state show the known features of the XRD patterns of the nematic phases of the pure cyanobiphenyl based dimers, *i.e.*, diffuse crescent-like scattering on the meridian in the small angle region (parallel to the orienting magnetic field) and diffuse crescent-like scattering on the equator in the wide-angle region collected at 112 °C and shown in Figure 15a. Similar to the dimer containing cyanobiphenyl units, this pattern does not change significantly at the phase transition to the low-temperature N_X_ phase. A representative diffraction pattern, collected at 80 °C, is shown in Figure 15b. Small angle intensities occur. θ-scans of the diffraction pattern in the N and N_x_ and the isotropic phases are shown in Figure 15c. As the small angle intensities persist in the isotropic phase, they are attributed to the average distance of the gold nanoparticles dispersed in the mixture. The distribution of the wide-angle scattering along χ in the N and N_x_ phases (plotted are the recorded intensities *vs.* χ (15°–25° 2θ)) is shown Figure 15d for data recorded at 112 °C and 80 °C. To enhance the visibility of the signal, the raw data was divided by the data collected in the isotropic phase at 135 °C. It is noticeable that the macroscopic orientation in the N_x_ phase is much larger than in the N phase, where there is hardly any increased orientation when compared to the isotropic phase. Beyond the small angle intensity, attributed to the gold nanoparticles, there is no indication of a transition to a smectic phase, even though the POM textural features, such as the fan-shaped texture, could suggest such a smectic-like structure formation.

**Figure 16.**
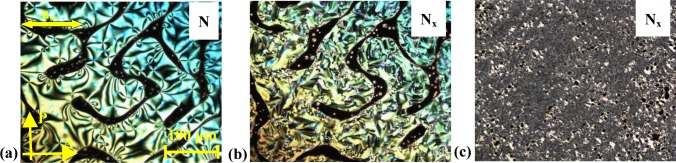
Textures of the binary mixture, **MGTP211** (15% (w/w) **4** in 85% **CB_C9_CB**), between untreated glasses observed in the same region upon cooling between crossed polarizers: (**a**) schlieren texture in the high-temperature nematic N phase at 113 °C; (**b**) fan-like texture of the low-temperature N_x_ phase at 100 °C; and (**c**) sheared texture of the low-temperature N_x_ phase at 84 °C.

Based on these results, the investigations were extended to the gold nanoparticle system, **4**, where the synthesis and the liquid crystal properties have been reported elsewhere [24]. The NP system, **4**, is characterized by particle diameters of ~10 nm; the LC groups are attached by an amino group to the gold nanoparticles; the spacer linking LC groups and nanoparticles contain a siloxane group, introduced in order to lower the glass transition temperatures and the viscosity of the system. Based on the previous results and for systematic reasons, a high concentration of **CB_C9_CB** in the mixture was targeted. The results of the investigation of the mixture, **MGTP211** (15% **4** in **CB_C9_CB** w/w), will be described. The POM studies and the calorimetric investigations evidenced the existence of an N–N_x_ phase sequence with textural and structural features similar to the pure cyanobiphenyl dimer, **CB_C9_CB**. The high-temperature phase could be easily identified as a nematic phase by its characteristic texture. At the phase transition to the N_X_ phase, a fine structured fan-shaped texture develops, shown in Figure 16a,b. The sheared texture contains a non-specific features, a small-scale fan-shaped texture and homeotropically oriented regions and some oily streaks, shown in Figure 16c.

**Figure 17.**
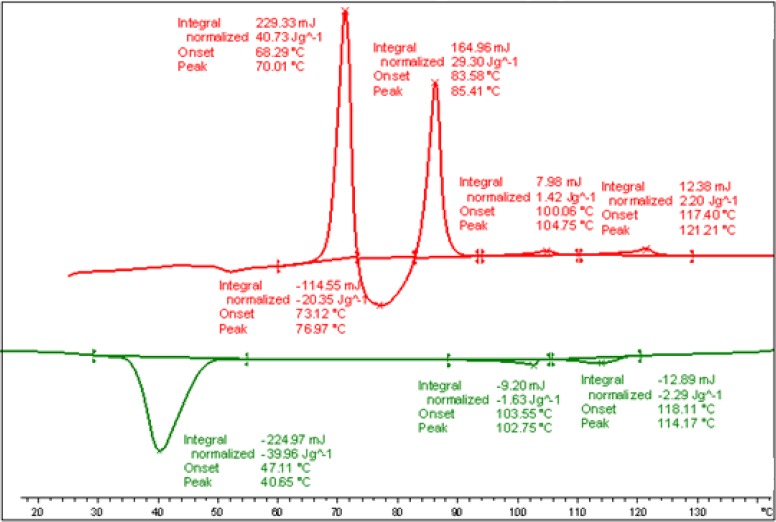
DSC studies (10 K/min) of the mixture, **MGTP211**; the values are taken from the first heating and cooling scans. Heating: Cr 85.4 (29.3) Nx 104.7 (1.42 ) N 121.2 (2.20) Iso. Numbers in brackets, transition enthalpies J·g^−1^. Cooling: Iso 114.2 (−2.29) N 102.7 (−1.63) N_x_ 47.1 (−39.96) Cr; the numbers in brackets are the transition enthalpies in J·g^−1^.

DSC measurements were conducted at varying heating and cooling rates of the samples of the binary mixture, **MGTP211** (15% (w/w) **4** in 85% (w/w) **CB_C9_CB**). The transition temperatures and the corresponding enthalpy values taken from the heating and cooling DSC scans (10 K/min) are shown in Figure 17. The surprising result is that the N_x_ phase formation has been stabilized; the composition with a 15% (w/w) addition of **4** forms an enantiotropic N_x_ phase, stable upon heating up to 104.7 °C.

**Figure 18.**
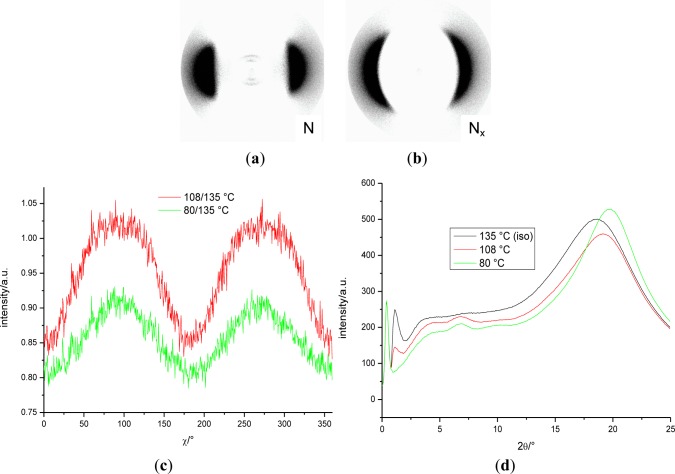
XRD patterns of an aligned sample of the mixtures, **MGTP211**, in a magnetic field (vertical) on cooling: (**a**) N phase at 108 °C; (**b**) N_x_ at 80 °C; (**c**) distribution of the wide-angle scattering along χ in the N and N_x_ phases; intensity *vs*. χ (15°–25° 2θ); (**d**) θ-scan of the diffraction pattern in the N and N_x_ phases; SAXS: *d* = 1.29 nm (108 °C), *d* = 1.29 nm (80 °C).

X-ray measurements were performed whilst applying a magnetic field of about 0.5 T on oriented samples of the binary mixture, **MGTP211**. The results show that the composition can be aligned by a relatively weak magnetic field. The results confirm the presence of two nematic phases. The X-ray patterns in the high-temperature nematic state show the known features of the XRD patterns of the nematic phases of the pure cyanobiphenyl or terphenyl dimers, *i.e.*, diffuse crescent-like scattering on the meridian in the small angle region (parallel to the orienting magnetic field) and diffuse crescent-like scattering on the equator in the wide-angle region, shown in Figure 18a; the data were recorded at 108 °C. Similar to a pure dimer containing cyanobiphenyl units, this pattern does not change significantly at the phase transition to the low-temperature N_X_ phase; an example is in Figure 18b of the data recorded at 80 °C. Figure 18c shows the distribution of the wide-angle scattering along χ in the N phase at 108 °C and N_x_ phases at 80 °C (plot: recorded intensity *vs.* χ (15°–25° 2θ); the values are divided by the data recorded in the isotropic phase at 135 °C, for better contrast). Though there seems to be some sharpening of the intensities at ~90°, parallel to the external magnetic field, at 80 °C, when compared to the nematic phase, overall, the differences are minimal. The θ-scan of the diffraction patterns in the N and N_x_ phases at 108 and 80 °C show no strong small angle intensities; the data is very similar to the pure **CB_C9_CB**.

The absence of the reflections for average distances of the large gold particles is attributed to their size. They are anticipated to be at smaller angles than what can be recorded with the equipment available. There is no indication of a transition to a smectic phase, even though the textural features, such as the fan-shaped texture and polygonal textures, could suggest a smectic-like structure, indicative of the limited use of the analysis of optical defect textures for the identification of the N_x_ phase formation.

## 3. Experimental Section

The synthesis of the materials has been reported elsewhere. X-ray diffraction experiments were performed on an MAR345 diffractometer with a 2D image plate detector (CuKα radiation, graphite monochromator, λ = 1.54 Å) (MAR Research, Hamburg, Germany). The samples were heated in the presence of a magnetic field using a home-built capillary furnace. Phase transitions were determined using a Mettler DSC822e stage (Mettler Toledo Intl. Inc. Switzerland) DSC with STARe software calibrated with indium in a nitrogen atmosphere calibrated against an indium standard (156.6 °C; 28.45 J·g^−1^; reported transition temperatures are the peak of the endotherm). Polarising microscopy studies were carried out using an Olympus BX51 polarising microscope (Olympus Corp., Tokyo, Japan). The microscope was equipped with a Mettler-Toledo FP900 heating stage (Mettler Toledo Intl. Inc., Greifensee, Switzerland).

## 4. Conclusions

In a systematic study, the dimer system, **CB_C9_CB**, was mixed with a laterally functionalized monomer, a structurally related laterally connected dimer system and LC nanoparticles, where the diameter of the gold particles was varied between ~3.3 and ~10 nm. In all the investigated materials, a nematic-nematic phase transition could be introduced, though at varying concentrations of the components. The compositions were characterised by POM, DSC and XRD investigations. The results show that it is possible to construct stable liquid crystal nanocomposites with materials having a metallic plasmonic response. It was found that, surprisingly, the N_x_ phase formation was not destabilized in mixtures with liquid crystalline gold nanoparticles; however, destabilization of the N_x_ phase formation occurred when mixing with a conventional laterally functionalized nematic monomer and a structurally related dimer. These results open up investigations of systems that combine the advantageous properties of nematic-nematic systems with those that have the properties of metallic nanoparticles.

## Figures and Tables

**Table 1.** Transition temperatures of the binary mixtures (w/w) of **1** with dimer **CB_C9_CB**, taken from the first DSC heating and cooling scans (10 K·min^−1^). Cr = Crystallie; N = Nematic; Nx = Nematic (x or tb); Iso = isotropic liquid.

Binary mixtures	1 % (w/w)	CB_C9_CB % (w/w)	Transition temperatures (°C)
**CB_C9_CB**	0	100	Cr 85.2 N_x_ 107.3 N 124.2 Iso; Iso 122.4 N 104.9 N_x_ 40.5 Cr
MGTP205	5	95	Cr 69.9 N_X_ 95.8 N 112.8 Iso; Iso 111.3 N 92.9 N_X_ 35.3 Cr
MGTP204	10	90	Cr 85.8 N 108.5 Iso; Iso 107.2 N 86.7 N_X_ 31.1 Cr
MGTP200	15	85	Cr 83.7 N 105.2 Iso; Iso 104.5 N 77.8 N_X_ 31.1 Cr
MGTP206	20	80	Cr 85.2 N 101.1 Iso; Iso 97.2 N 72.7 N_X_ 35.3 Cr
MGTP207	25	75	Cr 84.7 N 96.1 Iso; Iso 90.9 N 70.0 N_X_ 29.9 Cr
MGTP201	30	70	Cr 69.6 N 90.9 Iso; Iso 86.5 N 62.1 N_X_ 34.2 Cr
MGTP202	40	60	Cr 68.4 N 83.8 Iso; Iso 80.3 N 51.4 N_X_ 34.3 Cr
MGTP203	50	50	Cr 66.5 N 78.7 Iso; Iso 74.6 N 31.7 Cr
**1**	100	0	Cr 54.5 N 73.3 Iso; Iso 71.8 N 2.06 X −24.6 Cr

**Table 2.** Transition temperatures of the binary mixtures (w/w) of **2** with the dimer, **CB_C9_CB**, taken from the first DSC heating and cooling scans (10 K·min^−1^).

Binary mixtures	2 % (w/w)	CB_C9_CB % (w/w)	Transitions temperatures (°C)
**CB_C9_CB**	0	100	Cr 85.2 N_x_ 107.3 N 124.2 Iso; Iso 122.4 N 104.9 N_x_ 40.5 Cr
**MGTP208**	15	85	Cr 86.7 N 112.0 Iso; Iso 110.3 N 88.1 N_X_ 41.4 Cr
**MGTP209**	25	75	Cr 86.7 N 108.8 Iso; Iso 106.4 N 83.7 N_X_ 41.2 Cr
**2^a^**	100	0	Tg −8.8 N 107.6 Iso; Iso 106.3 N −14 Tg

a Values for the heating scan are taken from the second heating scan.
